# Difference Between Users and Nonusers of a Patient Portal in Health Behaviors and Outcomes: Retrospective Cohort Study

**DOI:** 10.2196/13146

**Published:** 2019-10-07

**Authors:** Jing Huang, Yong Chen, J Richard Landis, Kevin B Mahoney

**Affiliations:** 1 Perelman School of Medicine University of Pennsylvania Philadelphia, PA United States; 2 Institute for Biomedical Informatics University of Pennsylvania Philadelphia, PA United States; 3 Center for Evidence-Based Practice University of Pennsylvania Philadelphia, PA United States; 4 Center for Pharmacoepidemiology Research and Training Perelman School of Medicine University of Pennsylvania Philadelphia, PA United States; 5 Applied Mathematics and Computational Science University of Pennsylvania Philadelphia, PA United States; 6 Richard J Fox School of Business and Management Temple University Philadelphia, PA United States

**Keywords:** health behavior, health status, health care providers, patient portals

## Abstract

**Background:**

Patient portals are frequently used in modern health care systems as an engagement and communication tool. An increased focus on the potential value of these communication channels to improve health outcomes is warranted.

**Objective:**

This paper aimed to quantify the impact of portal use on patients’ preventive health behavior and chronic health outcomes.

**Methods:**

We conducted a retrospective, observational cohort study of 10,000 patients aged 50 years or older who were treated at the University of Pennsylvania Health System (UPHS) from September 1, 2014, to October 31, 2016. The data were sourced from the UPHS electronic health records. We investigated the association between patient portal use and patients’ preventive health behaviors or chronic health outcomes, controlling for confounders using a novel cardinality matching approach based on propensity scoring and a subsequent bootstrapping method to estimate the variance of association estimates.

**Results:**

Patient-level characteristics differed substantially between portal users, comprising approximately 59.32% (5932/10000) of the cohort, and nonusers. On average, users were more likely to be younger (63.46 years for users vs 66.08 years for nonusers), white (72.77% [4317/5932] for users vs 52.58% [2139/4068] for nonusers), have commercial insurance (60.99% [3618/5932] for users vs 40.12% [1632/4068] for nonusers), and have higher annual incomes (US $74,172/year for users vs US $62,940/year for nonusers). Even after adjusting for these potential confounders, patient portal use had a positive and clinically meaningful impact on patients’ preventive health behaviors but not on chronic health outcomes.

**Conclusions:**

This paper contributes to the understanding of the impact of patient portal use on health outcomes and is the first study to identify a meaningful subgroup of patients’ health behaviors that improved with portal use. These findings may encourage providers to promote portal use to improve patients’ preventive health behaviors.

## Introduction

### Background

Chronic conditions are the leading causes of death and disability and key drivers of total health care costs in the United States [1-3]. According to the Centers for Disease Control and Prevention, 7 of the top 10 causes of death in the United States were chronic conditions. As of 2012, more than half of all the adults in the United States suffered from at least one chronic condition, with approximately 25% of adults having 2 or more chronic conditions; moreover, these proportions are expected to increase in the next decade [4-6]. Chronic conditions also account for the vast majority of health spending in the United States. Each year, 86% of the nation’s total health care costs were for patients with at least one chronic condition and 71% were for patients with multiple conditions. In regard to public insurance, treatment of chronic conditions accounts for an even higher proportion of spending: 96% for Medicare and 83% for Medicaid [7-9].

From a public health perspective, primary prevention and screening for early-stage chronic conditions are considered the best strategies to prevent chronic conditions and to facilitate detection of disease at a milder stage of severity, thus incurring lower medical costs. Many chronic conditions could be prevented, delayed, or alleviated through simple lifestyle changes and other noninvasive interventions. In addition, it has been shown that preventive screening tests can reduce the death and comorbidity rates related to chronic conditions. For example, it has been reported that increased screening rates can reduce colorectal cancer deaths by 15% to 33% [10,11], and blood pressure checks for cardiovascular risk assessment can reduce cardiovascular morbidity of the population [12-14].

In the past few years, patient portals have been widely used in health care systems and have gained increasing attention for their potential values in improving health [15-22]. As a secure internet-based channel that provides patients with convenient access to personal health records, management of health services, and communication with health professionals, patient portals are considered as promising tools in promoting patient health outcomes, especially for chronic conditions, through promoting preventive behaviors, for example, taking screening tests, improving patient engagement in health outcomes, and facilitating self-management of chronic conditions [17,20,21].

Recently, several studies have investigated a variety of research questions related to portal use, including the characteristics of early portal users [23,24], the information being communicated through portals [25], patients’ and clinicians’ attitudes toward the use of patient portals [26], and the impact of patient portals on medication adherence and patient follow-up behaviors [20,27]. These important studies have contributed to the understanding of the role of patient portals in promoting communications between patients and health care providers in primary care and reducing health care cost by providing remote consultation as a low-cost alternative to physical office visits. Despite these successes, evidence is still scarce on whether the use of patient portals can ultimately improve patient health outcomes or modify patient preventive health behaviors positively [28,29].

### Significance of This Study

A major challenge in evaluating the impact of the portal use on patient health outcomes using electronic health record (EHR) data is the inherent selection bias. Specifically, the use of patient portals can be related to patients’ or health care providers’ characteristics, which may confound the associations between the portal use and patient health outcomes. Naïve regression analysis of the health outcomes on patient portal use is biased upward if patients adopted the portal because they have higher health motivations and better strategies of managing health conditions.

As patient portals are expected to improve patient experience and engagement, we hypothesized that portal use may have a clinically meaningful impact on patients’ preventive health behaviors, such as annual flu vaccination and blood pressure checks. Such an impact could be because of the benefit from better patient experience and engagement. Specifically, in this paper, we evaluated the impact of patient portal use on patients’ preventive health behaviors and chronic health outcomes using EHR data from a well-defined patient cohort within the University of Pennsylvania Health System (UPHS).

## Methods

### Study Population

We conducted a retrospective observational cohort study using data from the Penn Data Store (PDS) [30]. There were 2 inclusion criteria for this study: (1) the patient was aged 50 years or older and (2) the patient had been seen by a Penn-employed primary care provider at least once within the time window from September 1, 2014, to October 31, 2016. The study population consisted of 10,000 patients randomly selected from the PDS. On the basis of these criteria, we assumed the study population was representative of the middle-aged patient population of greater Philadelphia. This study was reviewed and approved by the institutional review board at the University of Pennsylvania.

UPHS is a diverse research and clinical care organization located in Philadelphia, Pennsylvania. Founded in 1993, it currently operates under the direction and auspices of Penn Medicine, a division of the University of Pennsylvania. A total of 6 hospitals, including the flagship Hospital of the University of Pennsylvania, with over 5000 clinical care providers in the Greater Philadelphia Area, constitute the UPHS. UPHS serves over a million unique patients a year. Although most of these patients are located in the 28 counties in Southeast Pennsylvania, Central and Southern New Jersey, and Delaware, as a nationally recognized leader in care, UPHS provides care to patients in all 50 states, the District of Columbia, Puerto Rico, Guam, and the Virgin Islands. The average patient age is 50 years, and 60% of the patients are female. UPHS serves a diverse patient population where 62% of the patients are white, 21% are black or African American, 3% are Hispanic, and 3% are Asian. The patient payer mix is 44% commercial, 41% Medicare, 14% Medicaid, and 1% other.

### myPennMedicine

**Figure 1:**
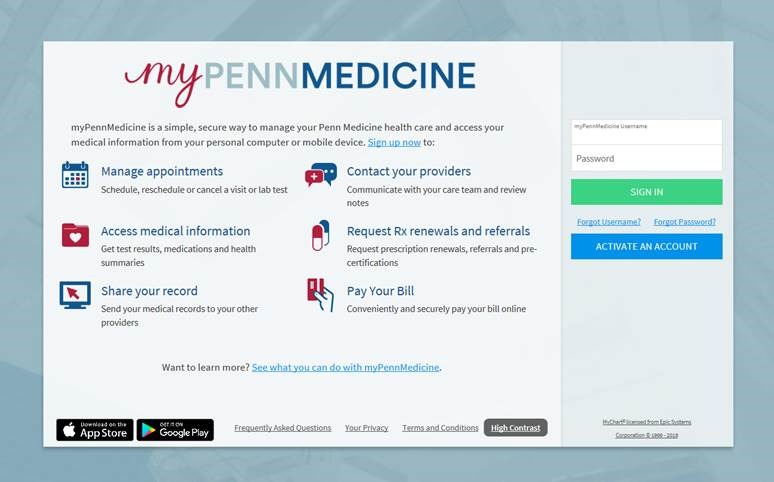
A screenshot of the myPennMedicine Web portal.

*myPennMedicine* is a Penn Medicine–branded version of Epic’s MyChart patient-facing electronic medical record. It is a patient portal that provides users with real-time information about medical records and test results, prescriptions, and appointments and other important health information. Patients may use the site to schedule appointments and laboratory tests, communicate with care teams, request prescription renewals and referrals, pay bills, and share records with other health care providers. It is available as a desktop Web portal and as an app for download from the Apple Store and Google Play. Patients must register, create an account, and log in to use these features. A screenshot of the *myPennMedicine* Web portal is shown in Figure 1. We defined patient portal users as patients who had registered for *myPennMedicine*. By May 2019, *myPennMedicine* had 591,784 unique and alive users. Among them, more than 66% had at least one activity in the past 365 days and approximately 45% and 28% had at least one activity in the past 90 and 30 days, respectively.

### Health Outcomes of Interest

Health information of the study population was extracted from the PDS. The preventive health behaviors were measured by 4 binary (yes or no) variables contained in the EHR: annual flu vaccination, blood pressure check, colorectal cancer screen, and lipid level screen. We also calculated an overall summary variable, hereafter referred to as the composite prevention score, as the sum of the 4 binary variables, ranging from 0 to 4 (higher is better). For patients’ chronic health outcomes, we studied 2 continuous and 2 binary variables from the EHR: systolic blood pressure, low-density lipoprotein, diabetes status (yes or no), and hypertension status (yes or no).

### Patient- and Provider-Level Characteristics

We also extracted demographics of the patients and their health care providers from the PDS. Our analyses included 5 patient-level characteristics—age (in years), annual income (in US $), sex, race/ethnicity (white, black, Hispanic, and others), and insurance type (commercial, Medicaid, and Medicare)—and 3 provider-level characteristics—sex, research type (faculty vs nonfaculty), and certification type (physician vs advanced care provider).

### Statistical Analysis

We evaluated the impact of the portal use on patient health outcomes by comparing the 4 preventive health behaviors and 4 chronic health outcomes between the portal users and nonusers. To control for potential confounders, including patient-level and health provider–level characteristics, we used a recently developed cardinality matching approach to match patient portal users to nonusers without replacement [31].

The cardinality matching procedure consists of 2 steps: balancing and pairing. First, this procedure maximizes the size of a match with prespecified requirements for balance on covariates, using integer programming. Specifically, we specified the differences in means of continuous covariates to be at most 0.05 SDs apart (moment balance) and required distributional balance on nominal covariates, without constraining users and nonusers to be matched within each category of each nominal covariate (fine balance) [32]. Then, with the groups determined and fixed, pairs were formed using minimum distance pair matching for a robust rank-based Mahalanobis distance computed based on the propensity score [31]. The major advantage of the cardinality matching approach, compared with traditional matching approaches, is that it finds the maximal number of matched samples (ie, the maximum cardinality) with balanced covariates in the 2 groups as a whole. Traditional matching algorithms find matched groups that are balanced for covariates at the same time as they find pairs that are close in their covariates. By doing this, typical algorithms do not usually find the largest number of matched samples that balance observed covariates. More importantly, the cardinality matching method was shown to have greater efficiency and lower sensitivity to unmeasured biases [31-33].

In the second step of cardinality matching, that is, to find the matched pairs using propensity score–based distance, there are multiple options of distance measures. The commonly used distances calculated from the propensity score include Euclidean distance [34,35], weighted sum of absolute differences [36,37], and Mahalanobis distance [38-40]. In our analyses, we used a rank-based Mahalanobis distance with a caliper for penalty violations on the propensity score to constitute a robust distance for matching [31]. The rank-based Mahalanobis distance reduces the influence of outliers in the matching, and the penalty for caliper violations ensures good balance on propensity scores.

After matching, we evaluated the association between the portal use and patient health outcomes by comparing the difference in means for continuous variables and difference in proportions for binary variables, and we used a bootstrap method to compute the SEs of the estimates [41]. Although the standard testing procedures, for example, paired *t* test and McNemar test, have been widely used in practice to test the statistical significance of the association after propensity score matching, it has been shown that such procedures can be misleading with underestimated variances [42,43]. Specifically, in addition to sampling variation, the variance of the estimators after matching should also account for the variability because of the estimation of the propensity score, the imputation of the covariates, and possibly also the order in which individuals are matched [44]. In addition, standard nonparametric bootstrap procedures based on resampling fail to be consistent in this context [42] as the standard bootstrap procedure fails to reproduce the distribution of the true matching function. To properly account for the variation in the matching procedure, a large sample approximation to the normal distribution using the variance estimate suggested by Abadie and Imbens [45] or a novel bootstrap approach [41] can be used. We have adopted the latter strategy to obtain the variances of the effect size estimates after matching.

## Results

### Patient- and Provider-Level Characteristics

Among the 10,000 patients extracted from the PDS, a total of 5932 patients were registered to use the *myPennMedicine* patient portal. After matching, we obtained 3465 pairs (ie, 6930 patients) of patient portal users and nonusers. Table 1 summarizes the patient- and provider-level characteristics between the portal users and nonusers before the propensity score matching. We found that the patient-level characteristics were significantly different between the 2 groups. Compared with nonusers, users were more likely to be younger (63.46 years for users vs 66.08 years for nonusers; *P*<.001) and have higher income (US $74,172 for users vs US $62,940 for nonusers; *P*<.001). The percentage of white race in users was substantially higher among portal users (72.77% (4317/5932) for users vs 52.58% (2139/4068) for nonusers; *P*<.001). The percentage of payment by commercial insurance was also substantially higher (60.99% (3618/5932) for users vs 40.12% (1632/4068) for nonusers; *P*<.001), and the percentage of payment by Medicare or Medicaid was substantially lower (Medicare: 34.91% (2071/5932) for users vs 48.72% (1982/4068) for nonusers; *P*<.001 and Medicaid: 3.49% (207/5932) for users vs 10.08% (410/4068) for nonusers; *P*<.001). The difference in sex between users and nonusers was not statistically significant. We did not find a significant difference in any provider-level characteristic between the 2 groups. The statistical significance was adjusted using Bonferroni correction to control type I error.

We applied the cardinality matching procedure using variables of both patient- and provider-level characteristics listed in Table 1. After matching, we compared the summary statistics of these variables between the patient portal users and nonusers, shown in Table 2, and the variables were balanced.

**Table 1:** Summary of patient- and provider-level characteristics in patient portal users and nonusers before propensity score matching.

Variables			Users	Nonusers	*P* value
**Patient characteristics^a^**					
	Age (years), mean (SD)		63.46 (9.22)	66.08 (11.37)	<.001
	Income (US $), mean (SD)		74,171.99 (29,150.87)	62,939.74 (30,017.67)	<.001
	Male, n (%)		2393 (40.34)	1592 (39.13)	.41
	**Race/ethnicity, n (%)**				
		White	4317 (72.77)	2139 (52.58)	<.001
		Black	1134 (19.12)	1564 (38.45)	<.001
		Hispanic	64 (1.08)	115 (2.83)	<.001
		Other	417 (7.03)	250 (6.14)	.08
	**Insurance type, n (%)**				
		Commercial	3618 (60.99)	1632 (40.12)	<.001
		Medicaid	207 (3.49)	410 (10.08)	<.001
		Medicare	2071 (34.91)	1982 (48.72)	<.001
		No insurance	36 (0.61)	44 (1.08)	<.001
**Physician characteristics, n (%)**					
	Faculty		2035 (34.31)	1217 (29.92)	<.001
	Physician		4466 (75.29)	3154 (77.53)	.01
	Male		2458 (41.44)	1661 (40.83)	.53

**^a^**Categorical characteristics are given as the percentage of patients, and numerical characteristics are given as mean (SD).

**Table 2:** Summary of patient- and provider-level characteristics in patient portal users and nonusers after propensity score matching.

Variables			Users	Nonusers	*P* value
**Patient characteristics^a^**					
	Age (years), mean (SD)		64.16 (9.97)	64.21 (10.00)	.89
	Income (US $), mean (SD)		67,128 (29,609.13)	66,807 (30,726.53)	.62
	Male, n (%)		1375 (39.68)	1380 (39.83)	.93
	**Race/ethnicity, n (%)**				
		White	2015 (58.15)	2002 (57.78)	.79
		Black	1168 (33.71)	1184 (34.17)	.85
		Hispanic	59 (1.70)	62 (1.79)	>.99
		Other	223 (6.44)	217 (6.26)	.87
	**Insurance type, n (%)**				
		Commercial	1745 (50.36)	1719 (49.61)	.86
		Medicaid	235 (6.78)	249 (7.19)	.74
		Medicare	1464 (42.22)	1475 (42.57)	.72
		No insurance	22 (0.64)	22 (0.63)	>.99
**Physician characteristics, n (%)**					
	Faculty		1112 (32.09)	1116 (32.21)	>.99
	Physician		2660 (76.77)	2661 (76.79)	.91
	Male		1396 (40.29)	1390 (40.12)	>.99

^a^Categorical characteristics are given as the percentage of patients, and numerical characteristics are given as mean (SD).

### Impact of Portal Use on Patient Health Outcomes

Table 3 presents the results from our analysis on matched patients. We found that patients’ preventive health behaviors were significantly associated with portal use. Specifically, the proportions of annual flu vaccination, blood pressure check, and lipid level screen were substantially higher in portal users compared with nonusers (odds ratios, OR=1.58, 1.13, and 1.50, respectively; *P*<.001 for all 3 outcomes). The average composite prevention score was also significantly higher among portal users compared with nonusers (mean difference=0.22; *P*<.001). We also found that the proportion of colorectal cancer screening test between portal users and nonusers was statistically significant (*P*<.001), but the OR was very close to 1. The statistical significance could be because of the large sample size, but the estimated OR did not show any clinically meaningful difference. We did not find any clinically meaningful difference between patient portal users and nonusers in chronic health outcomes. In the Multimedia Appendix 1, we visualized the difference in the percentages of annual flu vaccination, blood pressure check, and lipid level screen and the mean of the composite prevention score between portal users and nonusers.

**Table 3:** Estimated difference of health outcomes between patient portal users and nonusers after propensity score matching.

Outcomes		n	Effect size	*P* value
**Prevention health behaviors**				
	Flu shot	6930	1.58 (1.30 to 1.70)^a^	<.001
	Blood pressure test	6930	1.13 (1.08 to 1.29)^a^	<.001
	LDL^b^ test	6930	1.50 (1.38 to 1.67)^a^	<.001
	Colorectal cancer test	6930	0.99 (0.82 to 1.21)^a^	<.001
	Composite prevention score (0-4)	6930	0.22 (0.18 to 0.26)^c^	<.001
**Chronic health outcomes**				
	Systolic blood pressure	5692	−1.19 (−2.00 to 0.03)^c^	.06
	LDL	3756	−2.34 (−3.65 to 1.20)^c^	.50
	Diabetes status	6930	0.98 (0.79 to 1.20)^a^	.92
	Hypertension status	6930	0.97 (0.84 to 1.12)^a^	.69

^a^Odds ratio (95% CI) for binary variables.

^b^LDL: low-density lipoprotein.

^c^Mean difference (95% CI) for continuous variables.

## Discussion

### Principal Findings and Comparison With Previous Work

We investigated the association between the use of patient portal and patient- or provider-level characteristics. We found large differences between the portal users and nonusers in patient-level characteristics but not in provider-level characteristics. As both patient- and provider-level characteristics can potentially confound the associations between portal use and patient health outcomes, we adjusted for these potential confounders by adopting a recently developed cardinality matching approach based on propensity scores. After matching, the patient- and provider-level characteristics between portal users and nonusers were found to be balanced. We then quantified the impact of the portal use on patient health outcomes by comparing 4 different preventive health behavior variables and 4 chronic health outcome variables between the 2 groups. To ensure valid CIs and *P* values for the effect size estimates, we adopted a novel bootstrap method to estimate correct variances of the estimated impact of the patient portal.

Using EHR data from UPHS, our study contributed independent evidence to the impact of patient portal use on health outcomes, especially on preventive health behavior outcomes. This new evidence can lead to a better understanding of the value of patient portals in promoting patient health care of the highest quality.

With the implementation of EHR systems in almost all large health care systems in the United States and the availability of patient portals in many health systems, quantifying the impact of the portal use on patient health outcomes is important to patients, health care providers, policy makers, and other stakeholders. Patient portals hold great promise in improving communication between health care providers and patients, improving patients’ ability for self-management, enhancing their experience, improving health outcomes, and reducing medical costs. Recently, several investigators have published studies on understanding patient portal usability and satisfaction among users [15-29]. Investigators have found that the use of patient portals has significantly improved patients' ability to contact their providers directly, bypassing the usual gatekeepers in the practice, such as office staff and nurses in primary care; moreover, patient portals have also increased the follow-up rate in treatment of Crohn disease [17,20,25,27]. However, until now, very few studies have investigated the impact of portals on health outcomes. A very recent study of patient portals on hospital outcomes found that patient portals might not change hospital outcomes, for example, 30-day readmissions, inpatient mortality, and 30-day mortality, by comparing hospitalized patients who used portals with those who did not [29].

As discussed in the study by Dumitrascu et al [29], an important area for future research was to investigate the impact of patient portal use on more immediate outcomes. Our study aimed to fill this important evidence gap by investigating the impact of portal use on patients’ preventive health behaviors and chronic health outcomes using EHR data from UPHS. We chose preventive health behaviors and chronic health outcomes because they arguably create the greatest burden in health care and drive increases in medical costs. On the basis of our study on 10,000 adults (aged 50 years or older) recruited from an urban primary care system at Philadelphia, our investigation revealed that the use of patient portals significantly promoted preventive health behaviors, for example, taking flu vaccination and colorectal cancer screening tests, but it did not improve chronic health outcomes, for example, diabetes and hypertension status. We also found that the patients who have used patient portals have significantly different characteristics compared with patients who have never used patient portals. These patient portal users were more likely to be younger, have higher income, and use commercial insurance.

In this paper, we quantified the impact of patient portal use on preventive health behavior and chronic health outcomes based on the effect sizes (CIs) and their clinical meaningfulness. Given the large sample size of this study, we do not interpret statistical significance as clinical significance. For example, the mean difference of systolic blood pressure between portal users and nonusers was 1.19 mm Hg, which is close to statistically significant at .05 level but not clinically meaningful. In addition, interpretation of the study results may require caution in inferring causality. It should be noted that the diabetes and hypertension status in our study refer to the patients having the disease or not. The diseases could develop before or after the portal usage, such that for a subset of the study sample, the disease variables may be considered as baseline patient demographics rather than health outcomes. In this study, the comparisons of these disease statuses between portal users and nonusers are evaluations of cross-sectional associations between the portal usage and the disease status. Moreover, the observed preventive health behaviors may also be associated with specified chronic health outcomes. To reduce the potential confounder effect of the disease status on the association between the portal usage and prevention health behaviors, we also conducted the same analysis in subgroups defined by patients with hypertension, patients without hypertension, patients with diabetes, and patients without diabetes. We compared the same outcome variables between portal users and nonusers, except for the variable that was used to define the subgroup, and we obtained the same conclusion in these subgroup analyses as in the analysis using the entire dataset. We observed a statistically significant difference between portal users and nonusers in preventive health behaviors but not in chronic health outcomes.

### Limitations

Our study also has a few limitations that deserve further investigation. First, the study focused on relatively healthy patients aged 50 years or older. The conclusion may not be applicable to younger patients or patients with severe disease. It would be interesting to see whether similar investigations conducted in different patient cohorts result in the same or different conclusions. Second, the time window for this study was constrained from 2014 to 2016. Future research with a longer time window could be conducted to study the temporal relationship between portal usage and the risk of developing chronic conditions. Moreover, the frequencies of portal usage among the portal users were unknown in this study. The estimated effects could be attenuated if the frequencies were very heterogenous across portal users. In this study, patient data with log-in statistics were not available to study the effect of portal usage frequency on health behaviors or outcomes.

### Conclusions

The introduction of patient portals is widespread, and their success in promoting communication between patients and health care providers in primary care and reducing health care cost is being documented. This study is among the first to demonstrate that patient portal use is positively associated with patient preventive health behavior outcomes but not with chronic health outcomes. These findings contribute to the understanding and quantification of the impact of patient portal use on patient health outcomes. Additional research is required to confirm these findings. A future research direction is to understand the longitudinal impact of portal use on the trajectory of chronic health outcomes, which can provide new insights to patients, health care providers, policy makers, and other stakeholders on how patient portals can ultimately improve chronic health conditions.

## Appendix

Multimedia Appendix 1 Bar plot of percentage or mean values of prevention health behaviors among portal users and nonusers. BP: blood pressure; LDL: low-density lipoprotein.

## Abbreviations

EHR: electronic health record
OR: odds ratio
PDS: Penn Data Store
UPHS: University of Pennsylvania Health System
