# Supplementary figures and images for: Difference Between Users and Nonusers of a Patient Portal in Health Behaviors and Outcomes: Retrospective Cohort Study
Source: J Med Internet Res. 2019 Oct 7;21(10):e13146. doi: 10.2196/13146 (PMC6914108; doi:10.2196/13146)

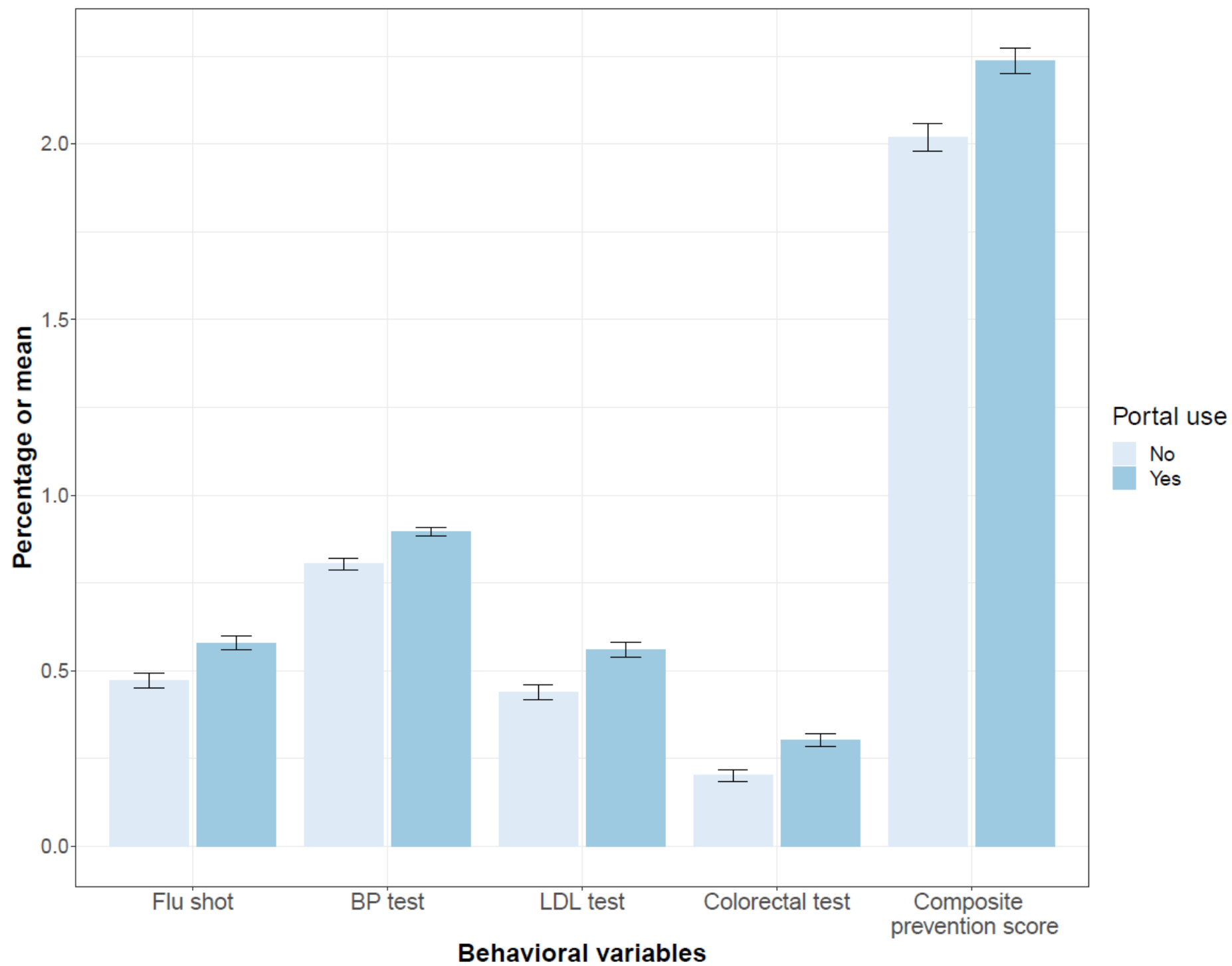

Supplement: Multimedia Appendix 1 [file jmir_v21i10e13146_app1.pdf]
